# Potential enhanced ability of giant squid to detect sperm whales is an exaptation tied to their large body size

**DOI:** 10.1186/1471-2148-13-226

**Published:** 2013-10-15

**Authors:** Lars Schmitz, Ryosuke Motani, Christopher E Oufiero, Christopher H Martin, Matthew D McGee, Peter C Wainwright

**Affiliations:** 1 W.M. Keck Science Department, Claremont McKenna College, Pitzer College, and Scripps College, Claremont, CA 91711, USA; 2 Department of Geology, University of California, Davis, CA 95616, USA; 3 Department of Biological Sciences, Towson University, Towson, MD 21252, USA; 4 Department of Environmental Sciences, Policy & Management, University of California, Berkeley, CA 94720, USA; 5 Department of Evolution and Ecology, University of California, Davis, CA 95616, USA

**Keywords:** Giant squid, Sperm whale, Eye size, Optical function, Scaling, Allometry, Adaptation, Exaptation

## Abstract

It has been hypothesized that sperm whale predation is the driver of eye size evolution in giant squid. Given that the eyes of giant squid have the size expected for a squid this big, it is likely that any enhanced ability of giant squid to detect whales is an exaptation tied to their body size. Future studies should target the mechanism behind the evolution of large body size, not eye size. Reconstructions of the evolutionary history of selective regime, eye size, optical performance, and body size will improve the understanding of the evolution of large eyes in large ocean animals.

## Introduction

The giant squid is an increasingly popular species for examining the optical functions and evolutionary drivers of large eyes in deep sea animals, with a series of papers published within the last 2 years [1-3]. Nilsson and colleagues [1] recently commented on a paper in which we explored the scaling relations of body and eye size in squid [2]. Our paper [2] itself was inspired by previous work of Nilsson and colleagues [3] who developed a functional model that allows exploration of vision in the deep sea. This model represented a major creative breakthrough in the field of animal sensory biology, without doubt paving the way for many future studies. Nilsson and colleagues introduced their new model with a case study on giant squid, one of the most enigmatic and charismatic animals populating the oceans today. Giant squid are elusive and thus very poorly known, yet optical modeling may indirectly illuminate some aspects of their biology. Nilsson and colleagues concluded [3] that the evolution of giant eyes may have been driven by predation, because very large eyes seemed uniquely suited for detecting large, approaching predators such as sperm whales. In our paper [2] we further explored this adaptive hypothesis and provided comparative eye size data for squid and a re-parameterization of Nilsson et al.’s model that casted doubt on swimming sperm whale predation driving giant squid eye evolution. The current contribution of Nilsson and colleagues [1] does not refute our data or observations. As we outline below, central to this interesting discussion is the methodological approach for testing adaptive hypotheses in evolutionary morphology.

## Discussion

Adaptations are traits that improve evolutionary fitness, originally shaped by natural selection for their current role [4]. Thorough tests of adaptive hypotheses require the integration of multiple lines of evidence [5,6]. A convincing phylogenetic argument for the case of an adaptation is achieved when selective regime, trait, and functional capacity of the trait evolve concordantly. When applied to the giant squid problem the following questions need to be addressed: a) when (i.e., along which lineages) did squid invade the mesopelagic realm and experienced predation from large oceanic predators?; b) when did relative eye size change?; and c) when did visual performance change? Given that sperm whales feed on many squid species other than giant and colossal squid [2], one would expect adaptive increases in eye size in several independent lineages.

Nilsson and colleagues provided an important piece to the puzzle in their original paper [3]: the modeling of functional capability of eyes depending on their size and the physical characteristics of the environments in which eyes operate. However, the phylogenetic lines of evidence that are necessary to make a strong case for adaptation are missing. The development of a functional mechanism by itself, even though important, is insufficient to support evolutionary adaptations.

In our paper [2] we revisited the conclusions of Nilsson and colleagues in order to better understand the possible adaptive significance of giant squid eyes. Our paper was not designed as a comprehensive test of the adaptive hypothesis but provided the much needed benefit of (1) a new data set on the scaling of squid eye diameter that places the relative eye size of giant squid in a comparative context and (2) some reconsideration of the values Nilsson and colleagues used to parameterize their model. Our work led to two points where we differ with the conclusions of Nilsson and colleagues, concerning whether giant and colossal squid have exceptionally large eyes and whether the model results were consistent with a unique functional advantage to those large eyes.

1. On the first issue, we found the diameter of giant and colossal squid eyes fall within 95% prediction intervals on the basis of our data from 87 smaller squid species. Our data show that giant and colossal squid eyes are not unusually large for squid of their body size. Our conclusion was thus different from that of Nilsson and colleagues who wrote in their Current Biology paper (penultimate paragraph of page 685): “Although other squid species generally have large eyes for their body size, the allometric growth factor for smaller squid is below 0.7 [26], making the eyes of giant and colossal squid unusually large even for squid.” Our data indicate that this last statement is incorrect. Yet, in the current contribution [1] Nilsson and colleagues again conclude that “the giant eyes of giant squid are indeed unexpectedly large” (from the title of their work). It appears the discussion has become a semantic argument around what is meant by ‘unexpectedly large’. Our data show that giant and colossal squid are large squid whose eye size follows the scaling pattern between body size and eye size in other squid. They do not have unexpectedly large eyes for their body size. But the authors find our scaling argument irrelevant, dismissing it for not providing a functional explanation for the large eyes of giant squid. Hence, Nilsson and colleagues do not really address our argument except to assert that it does not matter. To the contrary, we contend below that the issues we raise with our scaling data are central to this discussion. We also note that we did not question the hypothesis of a ‘law of diminishing returns’ but caution to interpret the presence of negative allometry as sufficient evidence. Eyes of both terrestrial and marine vertebrates scale with negative allometry and this observation has been linked to the scaling of brains [7]. It will be difficult to test this hypothesis as there are many possible competing explanations for the observed pattern, but we hope this question will be addressed in future studies.

2. On the second issue, in the present contribution Nilsson and colleagues build upon our re-parameterization of the original parameterization of their model by adding a new element. In doing so they reinstated their original conclusion that giant squid eyes are unusually good at seeing the glow of bioluminescence produced by swimming sperm whales. However, it seems somewhat optimistic to move forward as though the giant squid problem is resolved with a third parameterization of the model. It has become clear that the results of the model and the implications are very sensitive to parameters used in the model and there will always be room for improvement. The new element in the model, an estimate of displaced water volume in which bioluminescence is triggered, is not exempt from this, especially given that it is not backed up by computational or experimental evidence. That being said, we strongly agree that these squid must sometimes be able to see the bioluminescence caused by swimming sperm whales. We also agree that big eyes improve performance in this task. Indeed, in our paper we emphasized that there are many benefits of large eyes to the visual performance of giant squid.

## Conclusions

Where does this leave us in the discussion of eye size evolution in giant squid? Enhanced light capture is a functional consequence of large eyes, but not necessarily the “explanation” for large eyes that Nilsson and colleagues argue they have provided. We believe that here lies the core issue in this discussion. As outlined above, the bar for a convincing adaptive argument of large eyes is much higher than showing how the structure functions.

Indeed, the simplest interpretation of the available data actually is that any enhanced ability of these squids to detect whales is an exaptation tied to their large body size – a performance trait that was carried along with the evolution of large body size. Most of the difference in eye size between these squid and other squid can be explained by the difference in body size. What needs explanation is not “why” giant squid have such large eyes, but “why” they have such large body size. Quantitatively, the answer to why the body became so large will provide most of the explanation for why they have such large eyes. Body size is an important ecological variable and multiple selective advantages appear to be tied to it [8] which should be considered in conjunction with optical benefits of the larger eyes.

As Nilsson and colleagues emphasize, giant squid have uniquely big eyes. But this is because they are uniquely big squid. While our conclusions could be overturned with more data on eye size, or especially with the addition of a phylogenetic perspective that would provide more precise reference taxa for comparison, for now this seems to be the most plausible explanation. The present contribution [1] does not refute our data or these observations, and hence we were surprised to see them dismissed as irrelevant. Our conclusions about whether giant squid eyes are exceptionally large were strongly supported by data, and we reserve some cautious skepticism for the work with the model as the inferences seem to depend heavily on its most recent parameterization.

However, the hypothesis put forward by Nilsson and colleagues is thought-provoking and we hope the field will continue to investigate this interesting problem. As the various parameterizations of the optical model have already shown, we can be confident that large eyes have a number of advantages to giant and colossal squid and perhaps can provide the unique defense against one of their primary predators that the authors argue for. But these functional arguments do not explain the ‘reasons for exceptionally large eyes in squid…’ (final paragraph of [3]). It will take detailed reconstructions of the evolutionary history of selective regime, eye size, optical performance, and body size to better understand the reason for large eyes in large ocean animals.

## Competing interests

The authors declare no competing interests.

## Authors’ contributions

The ideas and concepts presented in this manuscript were developed by LS, RM, CEO, CHM, MDM, and PCW. LS and PCW wrote the manuscript, which was read and approved by all authors.
